# *Agrobacterium-*mediated genetic transformation of *Coffea arabica *(L.) is greatly enhanced by using established embryogenic callus cultures

**DOI:** 10.1186/1471-2229-11-92

**Published:** 2011-05-19

**Authors:** Alessandra F Ribas, Eveline Dechamp, Anthony Champion, Benoît Bertrand, Marie-Christine Combes, Jean-Luc Verdeil, Fabienne Lapeyre, Philippe Lashermes, Hervé Etienne

**Affiliations:** 1 Centre de Coopération Internationale en Recherche Agronomique pour le Développement - Département des Systèmes Biologiques (CIRAD-BIOS). UMR-RPB (CIRAD, IRD, Université Montpellier II), 911 Avenue Agropolis, BP 64501, 34394 Montpellier, France; 2 IRD - Institut de Recherche pour le Développement, UMR RPB (CIRAD, IRD, Université Montpellier II), 911 Avenue Agropolis, BP 64501, 34394 Montpellier, France; 3 CIRAD-BIOS, MRI, UMR-DAP, Plant cell imaging platform (www.PHIV.cirad.fr), Avenue Agropolis, 34398 Montpellier, Cedex 5, France

## Abstract

**Background:**

Following genome sequencing of crop plants, one of the main challenges today is determining the function of all the predicted genes. When gene validation approaches are used for woody species, the main obstacle is the low recovery rate of transgenic plants from elite or commercial cultivars. Embryogenic calli have frequently been the target tissue for transformation, but the difficulty in producing or maintaining embryogenic tissues is one of the main problems encountered in genetic transformation of many woody plants, including *Coffea arabica*.

**Results:**

We identified the conditions required for successful long-term proliferation of embryogenic cultures in *C. arabica *and designed a highly efficient and reliable *Agrobacterium tumefaciens*-mediated transformation method based on these conditions. The transformation protocol with LBA1119 harboring pBin 35S GFP was established by evaluating the effect of different parameters on transformation efficiency by GFP detection. Using embryogenic callus cultures, co-cultivation with LBA1119 OD_600 _= 0.6 for five days at 20 °C enabled reproducible transformation. The maintenance conditions for the embryogenic callus cultures, particularly a high auxin to cytokinin ratio, the age of the culture (optimum for 7-10 months of proliferation) and the use of a yellow callus phenotype, were the most important factors for achieving highly efficient transformation (> 90%). At the histological level, successful transformation was related to the number of proembryogenic masses present. All the selected plants were proved to be transformed by PCR and Southern blot hybridization.

**Conclusion:**

Most progress in increasing transformation efficiency in coffee has been achieved by optimizing the production conditions of embryogenic cultures used as target tissues for transformation. This is the first time that a strong positive effect of the age of the culture on transformation efficiency was demonstrated. Our results make *Agrobacterium*-mediated transformation of embryogenic cultures a viable and useful tool both for coffee breeding and for the functional analysis of agronomically important genes.

## Background

Genome sequencing of important crop plants, such as wheat, sugarcane, tomato, potato, banana, eucalyptus, cacao and coffee, has already been completed or is in progress [1]. The resulting information opens significant new challenges in plant biology, including determining the function of predicted genes and introducing new desirable traits in pre-existing outstanding genotypes by genetic engineering in a shorter time. Shortening the time required is particularly attractive for the improvement of woody species [2]: pedigree selection programs in *Coffea arabica*, a preferentially autogamous woody species, often last 25 years. In response to an unexpected threat, genetically transformed plants could thus provide a satisfactory solution, all the more since in a few years, coffee genome sequencing [3] will enable identification of genes coding for very important agronomical traits linked to disease resistance and/or abiotic stresses.

*Agrobacterium*-mediated transformation is a widely used and powerful tool for introducing foreign DNA into many plant species. It has several advantages over physical transformation methods including its tendency to generate single or a low copy number of transgenes with defined ends and preferential integration into transcriptionally active regions of the chromosomes [4]. *Agrobacterium*-mediated DNA delivery has become a powerful tool in functional genomics as it is the most reliable way to assess gene function by generating gain-of-function or loss-of-function mutants [5]. However, for many important crop plants, including most woody species, a method for genetic transformation has either not yet been established or is still laborious and inefficient. For such species, higher throughput transformation systems are needed to be able to fully benefit from the rapid development of plant genomics for basic research and for the design of new genetic improvement strategies including both conventional breeding and genetic transformation.

There are very few examples of the introduction of genes of agronomic interest in Arabica coffee. Leroy et al. [6] regenerated transgenic coffee plants carrying the *CRY1-AC *gene from *Bacillus thuringiensis*, which is effective against the coffee leaf miner. Ogita et al. [7] obtained transgenic coffee plants with suppressed caffeine synthesis using RNA interference (RNAi) technology through inhibition of a theobromine synthase gene (*CaMXMT1*). Genetic transformation of coffee has been achieved using several types of explants (leaves, embryogenic calli, somatic embryos, protoplasts) and different approaches including *A. tumefaciens-*mediated transformation [6-9], *A. rhizogenes-*mediated transformation [10,11] and biolistic gene delivery [12,13]. The recovery of transgenic plants appears to be easier with *Coffea canephora *species than with *C. arabica *[14-16]. However the protocols available so far, which mostly use *A. tumefaciens*, are not reproducible and transformation efficiency is very low (less than 1%), thus seriously limiting their potential routine use. Today, embryogenic callus derived from leaf tissues is the most widely used target tissue for the genetic transformation of *C. arabica *[7,9,17]. However the induction of embryogenic tissues in *C. arabica *takes longer and is more difficult than in the other cultivated species *C. canephora*. The limited availability of embryogenic tissues, together with the low transformation efficiency of this type of tissue, is one of the main limitations to genetic transformation in coffee. Each round of a transformation experiment requires a new and uncertain process of production and selection of embryogenic calli that takes around eight months.

For these reasons, defining the conditions for successful long-term proliferation of embryogenic callus is a decisive step towards the large-scale, continuous production of target tissues for genetic transformation. Embryogenic cultures have already been used to establish reliable and efficient genetic transformation procedures for different woody species including *Prunus *[18,19], grapevine [20,21], rubber tree [22] and chestnut tree [23]. To date, embryogenic callus cultures maintained in liquid or semi-solid medium have never been used as target tissues for genetic transformation of the two cultivated species *C. arabica *and *C. canephora*. However, high-throughput methods for coffee propagation based on the use of established embryogenic cultures have already been developed by our team [24,25] and are currently used for the commercial diffusion of elite F1 hybrid varieties [26,27]. Such systems could also provide suitable support for the high-throughput regeneration of coffee transgenic varieties.

In the present work, we describe for the first time in the *C. arabica *species a highly efficient and reliable *Agrobacterium*-mediated transformation protocol that allows the generation of thousands transgenic coffee trees from multiple independent transformation events.

## Results

### Explant and co-cultivation conditions

**Figure 1:**
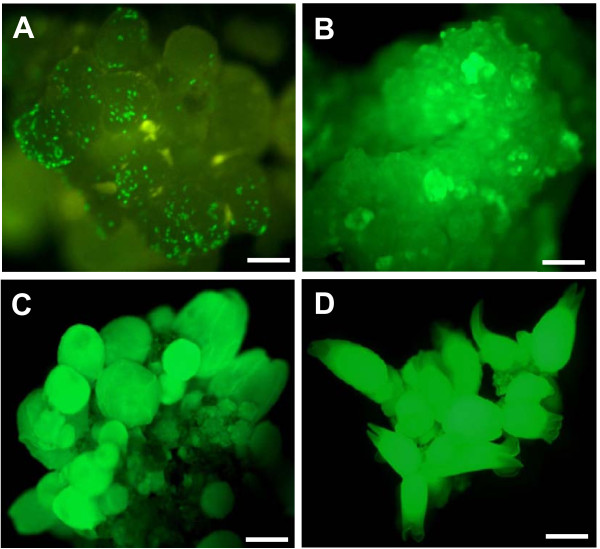
**GFP fluorescence in embryogenic callus cultures transformed with *A. tumefaciens *and in derived somatic embryos**. Transient expression in embryogenic callus 5 days after co-cultivation, bar scale = 50 μM (A). Stable expression in embryogenic callus culture 30 days after co-cultivation, bar scale = 50 μM (B). Stable expression in globular and heart stage 6 months after co-cultivation, bar scale = 100 μM (C). Stable expression in torpedo mature somatic embryos 7 months after co-cultivation, bar scale = 500 μM (D). Images were obtained using a Leica MZ Fluo III (optic 0.63 Zeiss) fluorescence microscope supplied with a DC 300F camera (Leica Microsystems, Welzlar, Germany) with plant GFP filter no. 3 from Leica: excitation wavelengths: 470-540 nm (BP), emission wavelengths: 525-550 nm (BP).

Different types of coffee embryogenic tissues were tested under our transformation conditions; GFP detection was performed 30 days after co-cultivation to confirm stable transformation events (Table 1). In this study, the detection of GFP expression was successfully used to monitor the transformation efficiency of all the parameters concerned. A low GFP expression level was detected in co-cultivated embryogenic calli and almost no expression was observed when embryogenic cell suspensions were used. On the other hand, embryogenic callus cultures were revealed to be an excellent support for genetic transformation, since significantly higher transformation efficiency (17%) was obtained with four-month-old proliferating embryogenic calli in the same co-cultivation conditions. Using epifluorescence analysis, strong GFP expression was detected in embryogenic callus cultures five and 30 days after co-cultivation and in derived somatic embryos at all ontogenetic stages (Figure 1).

**Table 1:** Effect of the type of target tissue on transformation efficiency

Type of target tissue	No. of co-cultivated calli	No. of transformed calli	Transformation efficiency (%)
Embryogenic callus	154	6	3.90 ± 5.80
Established embryogenic cell suspension (4 months)	192	4	0.02 ± 0.03
Established embryogenic callus culture (4 months)	240	41	17 ± 0.02

*Transformation efficiency was estimated by the proportion (p) of transformed calli (p = x/n), where x is the number of transformed calli and n the number of co-cultivated calli. A 3 δ confidence limit for binomial distribution was calculated using the formula  with a level of confidence of 99%

Other co-cultivation parameters that have been shown to be decisive in establishing an efficient *A. rhizogenes*-mediated transformation protocol [10] were tested to establish co-cultivation conditions with *A. tumefaciens *using embryogenic callus cultures. First, the efficiency of two different concentrations of bacterial suspension in enhancing genetic transformation with embryogenic callus cultures was compared (Table 2). Using an undiluted *A. tumefaciens *suspension with an OD_600 _of between 0.6 and 0.7 proved to be slightly better than a diluted bacterial solution (1/10). Similarly, we observed a slight increase in transformation efficiency by reducing the co-cultivation temperature from 27°C to 20°C (Table 2). Another advantage is that the lower temperature prevented bacterial overgrowth and made the decontamination process easier.

**Table 2:** Effect of different co-cultivation factors on transformation efficiency using established embryogenic callus cultures

Co-cultivation factors	Treatments	No. of co-cultivated calli	No. of transformed calli	Transformation efficiency (%)
*Agrobacterium *conc. (OD_600_)	1 (OD_600 = _0.6)	80	10	12.5 ± 11.1
	1/10	102	2	1.9 ± 4.1
Co-cultivation temperature (°C)	20	215	10	4.7 ± 4.3
	27	350	2	0.6 ± 1.2

*The transformation efficiency was estimated by the proportion (p) of transformed calli (p = x/n), where x was the number of transformed calli and n the number of co-cultivated calli. A 3 δ confidence limit for binomial distribution was calculated using the formula  with a level of confidence of 99%

### Effect of the composition of the embryogenic proliferation culture medium on subsequent transformation

**Figure 2:**
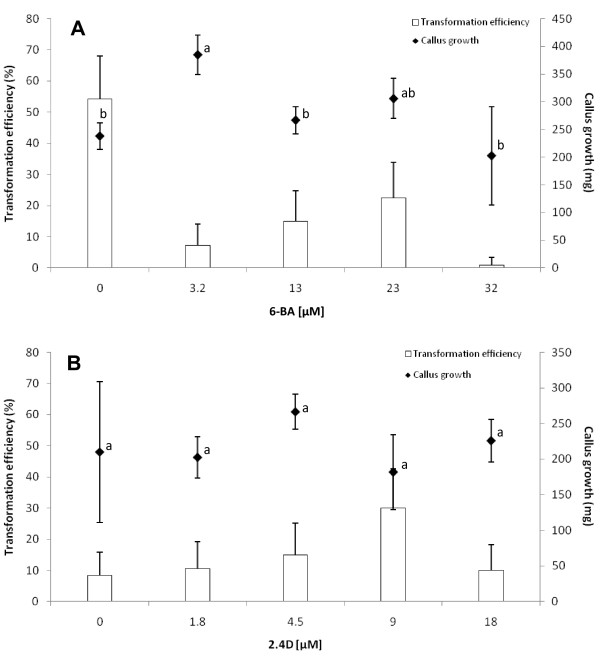
**Effect of 6-BA and 2,4-D concentrations in the proliferation medium on growth intensity and transformation efficiency**. All the 6-BA concentrations were tested in the presence of 4.52 μM 2,4-D and all the 2,4-D concentrations were tested in the presence of 12 μM 6-BA. Callus growth was measured by the difference between the final and initial weight of embryogenic cultures after a 4 week proliferation cycle. The initial weight was calibrated at 120 ± 10 mg. Each datum corresponds to the mean ± SD of 4 measurements. Confidence interval is indicated by vertical bars. We performed an ANOVA followed by the Tukey HSD test to identify significant differences between the means of all treatments. Values with different letters are significantly different at P ≤ 0.05. Transformation efficiency was assessed by observing GFP epifluorescence 6 weeks after the end of co-cultivation. Transformation efficiency was analyzed by the proportion (p) of transformed calli (p = x/n), where x was the number of transformed calli and n the number of co-cultivated calli. A 3 δ confidence limit for binomial distribution was calculated using the formula  with a level of confidence of 99%. All the transformation experiments were conducted independently in four replicates each comprising 60 calli (240 calli/ hormonal combination).

The composition of the nutritive medium used for embryogenic callus proliferation prior to genetic transformation can directly influence the physiological status of the embryogenic callus and hence the success of transformation. We consequently tested the effect of different salt concentrations and auxin to cytokinin ratios on the intensity of callus growth (on which the availability of tissues suitable for transformation depends) and on transformation efficiency. Successful transformation was only obtained with the diluted MS/2 and MS/4 salt concentrations (Table 3). The latter gave significantly higher transformation efficiency, but the growth of the embryogenic callus was seriously affected by this highly diluted salt concentration: growth was 2 to 2.5 times lower than at other salt concentrations. On the other hand, MS and 1.5 MS media rich in salts were not appropriate for coffee transformation. The MS/2 salt concentration that enabled efficient embryogenic callus proliferation and subsequently efficient transformation was thus selected for further transformation experiments. Regarding the auxin to cytokinin ratio in the proliferation medium, we first observed that 6-BA had a strong negative effect on genetic transformation, as very efficient transformation was obtained without exogenous cytokinin (Figure 2A). However, it should be noted that 6-BA was not needed to sustain embryogenic callus proliferation, as acceptable growth intensity over three sub-cultures was obtained without it. Like cytokinin, the concentration of 2,4-D had a weak effect on the growth of the embryogenic callus (Figure 2B). A relatively high auxin concentration (9 μM 2,4-D) in the embryogenic callus proliferation medium led to the highest transformation efficiency, confirming the positive effect of an auxinic environment on the pre-culture medium prior to coffee transformation.

**Table 3:** Effect of mineral salt concentrations in the embryogenic culture proliferation medium on callus growth and transformation efficiency

Mineral salt concentration in the ECP proliferation media	Callus growth (mg/month)	No. of co-cultivated calli	Transformation efficiency (%)
MS/4	103 ± 20.7 b*	87	15.6 ± 6.6**
MS/2	267 ± 24.1 a	160	10.5 ± 14.1
MS	254 ± 60.5 a	100	0
1.5MS	217 ± 53.6 a	160	0

* Callus growth was measured by the difference between the final and initial weight of embryogenic cultures after a 1 month proliferation cycle. The initial weight was calibrated at 120 ± 10 mg. Each datum corresponds to the mean ± SD of 4 measurements. We performed an ANOVA followed by the Tukey HSD test to identify significant differences between the means of all treatments. Values with different letters are significantly different at P ≤ 0.05. ** Transformation efficiency was analyzed by the proportion (p) of transformed calli (p = x/n), where x is the number of transformed calli and n the number of co-cultivated calli. A 3 δ confidence limit for binomial distribution was calculated using the formula  with a level of confidence of 99%. Each value is the mean of 4 replicates.

### Effect of the phenotype of the embryogenic culture

**Figure 3:**
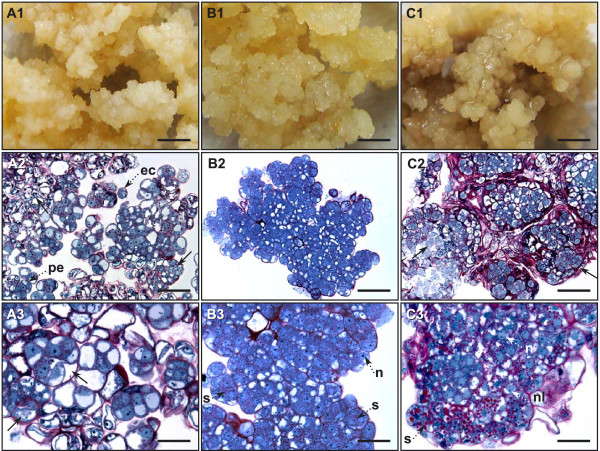
**Morphological and histological appearance of the different callus phenotypes observed in maintained embryogenic callus cultures assayed for coffee genetic transformation**. Whitish type embryogenic callus: morphology, scale bar = 2 mm (A1) and histological aspect (A2), (A3). Note the very loose structure of the callus and the presence of proembryos (pe), isolated embryogenic cells (ec) along with abundant degenerating cells and proembryos (arrow), scale bar = 73 μM (A2); note the segmentation and degenerating process of proembryos (arrow) and the low-density cytoplasm of all cells, scale bar = 36.5 μM (A3). Yellow type embryogenic callus: morphology, scale bar = 2 mm (B1) and histological appearance (B2), (B3); embryogenic callus comprising small cell aggregates similar to proembryogenic masses (PEMs); note the high homogeneity of the tissues, scale bar = 73 μM (B2); note the high nucleus cytoplasm ratio of all cells, the voluminous central nucleus (n) and the numerous small starch grains (s) around the nucleus; note the very dense cytoplasm rich in soluble and reserve proteins (blue staining) in all the cells, scale bar = 36.5 μM (B3). Gray type embryogenic callus: morphology, scale bar = 2 mm (C1) and histological appearance (C2), (C3); note the heterogeneous callus structure comprising a mix of degenerating tissues (on the left) and active (on the right) and intermediate areas, scale bar = 73 μM (C2); most of the cells are vacuolated with a non-central nucleus (n), a small nucleolus (nl) and less abundant but bigger starch grains (s), scale bar = 30 μM (C3).

**Figure 4:**
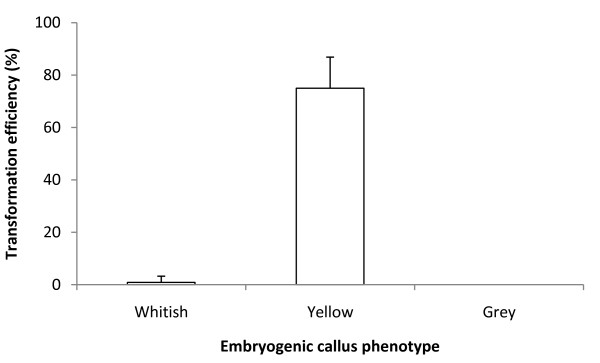
**Transformation efficiency depending on the phenotype of maintained embryogenic callus cultures**. Transformation efficiency was assessed by observing GFP epifluorescence 6 weeks after the end of co-cultivation. Transformation efficiency was estimated by the proportion (p) of transformed calli (p = x/n), where x is the number of transformed calli and n the number of co-cultivated calli. A 3 δ confidence limit for binomial distribution was calculated using the formula  with a level of confidence of 99%. Confidence interval is indicated by vertical bars. For each callus phenotype, all the transformation experiments were conducted independently in three replicates comprising 40 calli (120 calli/phenotype).

In spite of the overall success of coffee transformation, we observed marked disparity for the same optimized conditions. Whereas in some jars almost all the callus produced hygromycin-resistant calli, in others, no resistant callus was obtained. We suspected that the variability observed in transformation was due to the existence of an additional effect related to heterogeneity within the embryogenic material. Careful observation of the embryogenic callus cultures enabled us to distinguish between three callus phenotypes in the same culture conditions. All the phenotypes were friable but some differences in color and texture were identified. These phenotypes, called whitish, yellow and gray, are shown in Figures 3A1, B1, C1. The gray phenotype was more compact than the yellow one, and the whitish one was looser and grew the fastest. The whitish phenotype was also the most frequent (60-70%) whereas the yellow and gray ones represented 25%-35% and 5%-10% of the population, respectively. Based on this observation, we started separating the calli into three batches according to their phenotype, and tested them for genetic transformation. Figure 4 shows that no transformation event occurred using the gray callus phenotype and less than 1% with the whitish one. On the other hand, very high transformation efficiency (75%) was achieved using the yellow friable calli, thus proving them to be highly suitable for the genetic transformation of coffee.

Histological studies revealed major differences between the three embryogenic callus phenotypes (Figure 3). The whitish phenotype (Figure 3A1) displayed a much looser structure comprising isolated embryogenic cells, along with small pro-embryos (always < 10 cells) [Figure 3A2]. A proembryo segmentation and degeneration process was observed, corresponding to the initiation and rapid degeneration of somatic embryogenesis events. Cells constituting this type of callus did not exhibit a dense cytoplasm (nor did those belonging to proembryos) but had small starch grains and the visible mitosis stages suggested rapid divisions and growth (Figure 3A3). The yellow callus (Figure 3B1) corresponded to a highly homogeneous tissue mainly comprised of small cell aggregates similar to proembryogenic masses (PEMs) (Figure 3B2). The cells exhibited a high nucleus cytoplasm ratio and a voluminous central nucleus, numerous small starch grains around the nucleus and a very dense cytoplasm rich in soluble and reserve proteins (Figure 3B3). The cell walls varied in thickness as is typically observed in embryogenic cells [28,29]. The gray phenotype (Figure 3C1) was much more heterogeneous and was made up of a mix of degenerating tissues and active intermediate areas (Figure 3C2). The cells were more vacuolated with a non-central nucleus, a small nucleolus and less abundant but bigger starch grains (Figure 3C3). All these observations are generally associated with degenerating tissues. Sub-culturing the different types of calli separately showed that each phenotype was maintained over the sub-cultures (data not shown). The stability of these embryogenic callus phenotypes offered an opportunity to select the transformation-competent yellow phenotype and easily eliminate the undesirable ones during the proliferation process aimed at building up stocks of highly competent embryogenic material with a view to genetic transformation experiments.

### Effect of the age of the culture on transformation efficiency

**Figure 5:**
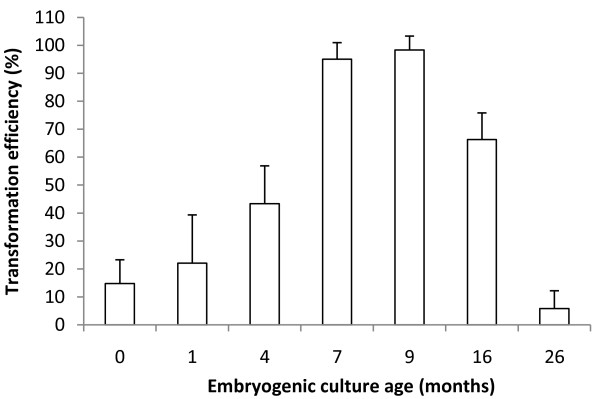
**Effect of the age of the embryogenic callus culture on *A. tumefaciens*-mediated transformation efficiency**. Transformation efficiency was assayed 6 weeks after the end of co-cultivation by GFP epifluorescence. The transformation efficiency was estimated by the proportion (p) of transformed calli (p = x/n), where x is the number of transformed calli and n the number of co-cultivated calli. A 3 δ confidence limit for binomial distribution was calculated using the formula  with a level of confidence of 99%. Confidence interval is indicated by vertical bars. All the transformation experiments were conducted independently in five to six replicates each comprising 40 calli for each culture age (200 to 240 calli/culture age).

Long-term maintenance of competent yellow type embryogenic callus was achieved by sub-culturing it every month on fresh gelled proliferation media. Embryogenic callus cultures with very variable proliferation durations (i.e. age of the culture) were transformed with *A. tumefaciens*. Figure 5 shows that the age of the embryogenic callus culture strongly affected transformation efficiency. Surprisingly, the best transformation efficiency (almost 100%) was achieved by using embryogenic callus cultures with at least seven months of proliferation, and much lower transformation efficiency (around 15%) was obtained with the primary embryogenic callus. Transformation efficiency gradually increased with the age of the embryogenic culture to reach maximum at seven and nine months. Transformation efficiency can be maintained at a high level over a long period of time, since 70% efficiency was still obtained with 16-month-old embryogenic cultures. However, 26-month-old embryogenic cultures had almost completely lost their ability for genetic transformation.

**Figure 6:**
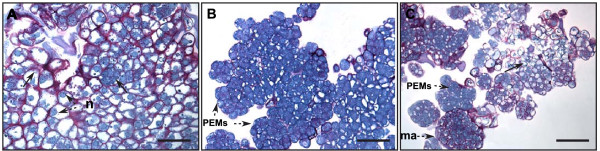
**Histological appearance of coffee embryogenic callus cultures as a function of the age of the culture**. Primary embryogenic callus produced on leaf explants; note the heterogeneous structure where degenerating (arrow) and active areas co-exist; the cells are characterized by a small non-central nucleus and low starch and cytoplasm soluble protein contents, scale bar = 36.5 μM (A). Seven-month-old embryogenic callus culture; note the highly homogeneous structure mainly comprising proembryogenic masses (PEMs) whose cells exhibit a high nucleus cytoplasm ratio and dense cytoplasm rich in soluble and reserve proteins, scale bar = 73 μM (B). Twenty-six month-old embryogenic callus culture; note the heterogeneous appearance of the mix of isolated cells, degenerating areas (arrow), masses oriented towards somatic embryogenesis (ma), along with areas of cells with protein rich cytoplasm (PEMs) similar to those in younger embryogenic cultures, scale bar = 73 μM (C).

Histological analysis revealed a change in the quality of embryogenic callus tissues depending on the age of the culture with no changes visible to the naked eye (Figure 6). Whereas the most homogeneous and active tissues were observed in seven and nine-month old embryogenic callus cultures that displayed the typical histological appearance of the yellow phenotype (Figure 6B), the histological appearance of the primary embryogenic callus (no proliferation period) and oldest embryogenic callus cultures was very different. Observation of primary embryogenic callus indicated a very heterogeneous structure (Figure 6A) with coexisting degenerating and active areas. The cells had a small nucleus and low starch and cytoplasm soluble protein contents. At the histological level, the old embryogenic cultures were much more heterogeneous than at seven months and appeared as a mix of isolated cells, proembryos and masses oriented towards somatic embryogenesis (Figure 6C). Other areas comprised cells with protein-rich cytoplasm similar to those in younger embryogenic cultures.

### Transgenic coffee plant regeneration and molecular analysis

**Figure 7:**
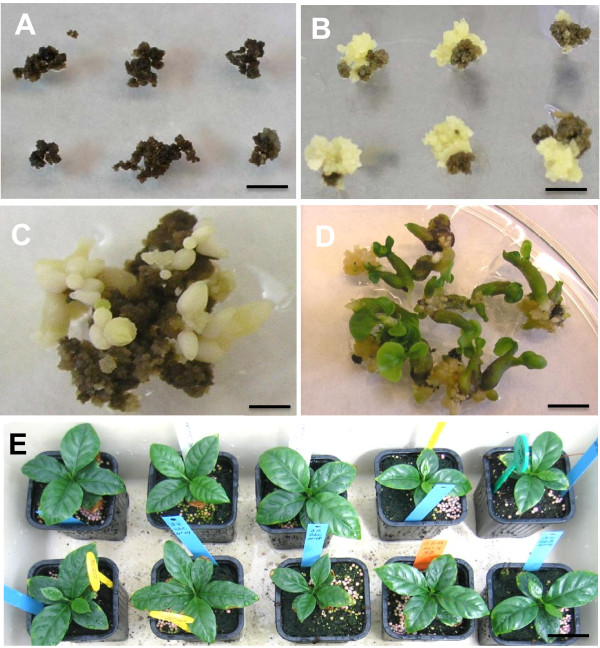
**Regeneration of transformed coffee plants from maintained embryogenic cultures**. Calli in selective media containing 100 mg.L^-1^hygromycin and 125 mg.L^-1 ^cefotaxime 4 months after co-cultivation with *A. tumefaciens *LBA1119 without plasmid, used as control (A). Regeneration of resistant calli in selective media containing 100 mg.L^-1^hygromycin and 125 mg.L^-1 ^cefotaxime 4 months after co-cultivation with *A. tumefaciens *LBA1119 carrying pMDC32; the yellow calli are resistant to hygromycin (B). Regeneration of torpedo-shaped somatic embryos 6 months after co-cultivation (C). *In vitro *plantlet development 8 months after co-cultivation (D). Transgenic plants in the greenhouse 12 months after co-cultivation (E).

Transgenic plants were regenerated using seven-month-old embryogenic cultures of the yellow phenotype, according to the optimum proliferation and co-culture conditions established in this work. A total of 560 calli were co-cultivated. The transfer of embryogenic calli to hygromycin selection medium led to rapid browning of both co-cultivated and untransformed calli used as control (Figure 7A), but two months later, a lot of resistant yellowish calli had grown on the surface of most of the necrotic calli (Figure 7B). After four months of hygromycin selection, among the 560 co-cultivated calli, a total of 462 produced independent yellow resistant calli (transformation efficiency = 82.5%). All the non-transformed cultures (negative control) turned brown and died during the hygromycin selection period and neither yellow calli nor embryo regeneration was observed. Each resistant callus line regenerated several putatively transformed somatic embryos on the hygromycin-enriched medium (Figure 7C). Almost all resistant torpedo-shaped mature embryos germinated and developed into whole plantlets (Figures 7D, E). Several thousand putatively transformed plants were regenerated. Among them, 120 plants from 60 independent transformation events (2 plants/transformation event), were chosen to be acclimatized in the greenhouse for further molecular analyses (Figure 7E).

**Figure 8:**
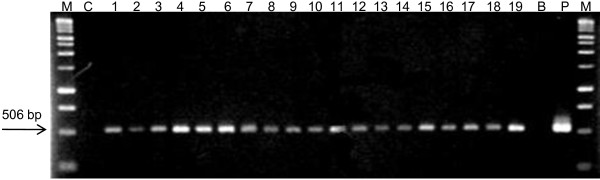
**PCR detection of the *HPTII *hygromycin resistance gene in transgenic coffee plants**. The plants were produced from 7-month-old embryogenic callus cultures co-cultivated with *A. tumefaciens *strain LBA1119 carrying out the pMDC32 binary vector. M - Molecular weight DNA markers (1 kb), C - untransformed coffee plant (control), *1-19 *transgenic coffee plants derived from independent transformation events, B - blank, PCR mix without DNA, P - plasmid. The arrow indicates the fragment corresponding to the *HPTII *gene (506 bp).

**Figure 9:**
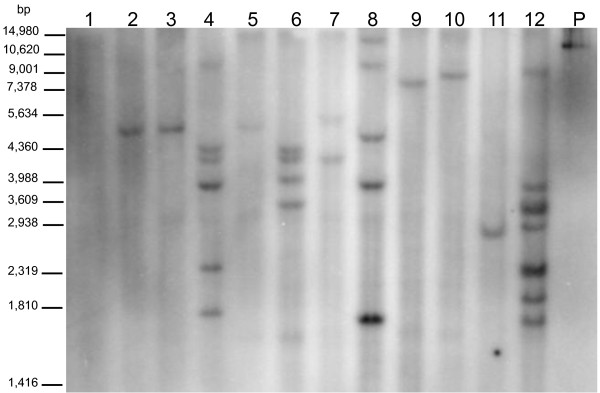
**Southern blot analyses of transgenic coffee plants**. Coffee DNA from leaf material was digested with *Eco*RI, electrophoresed and probed with a ^32^P labeled *HPTII *gene fragment. The number of bands reflects the number of transgene insertion sites. Lane 1, untransformed coffee (control). Lanes *2-12*, transgenic plants from independent transformation events. P - plasmid control (pMDC32). The size of the markers is indicated on the left.

PCR analysis was performed to detect the *HPTII *hygromycin resistance gene in the DNA of 60 putatively transformed plants derived from independent transformation events (1 plant/transformation event). Bands indicating the presence of the *HPTII *gene were systematically detected in the analyzed plants (Figure 8) indicating the high efficacy of hygromycin selection. No band was observed in untransformed plants or in the blank negative control. Southern blot analysis was performed to determine the number of insertion sites of the *HPTII *gene into the coffee genome (Figure 9). Eleven plants from independent transformation events were analyzed. Five of them presented one transgene insertion site (event n° 2, 3, 9, 10, 11). The others presented multiple copy insertion sites: two (n°5 and 7), between four and seven (n°4, 6, 8, 12).

## Discussion

In spite of an increasing number of successes, transgenic plant production is still difficult and limited to a small number of species and genotypes. This is particularly true of woody species, as these are often recalcitrant to genetic transformation. Chevreau [30] reported that only three fruit woody species were distinguished by a large number of successfully transformed genotypes: more than 10 species of *Citrus *[31], 40 genotypes of apple tree [32], and 35 of grapevine [33]. For the majority of species, the efficiency of the methods used remains variable and weak. However, efficient methods have been described in a number of woody crops. One category of methods consists in directly transforming zygotic embryos or young seedlings (epicotyls, hypocotyls) to rapidly obtain transformed organogenic calli and derived plantlets; this is the case in *Allocasuarina *[34], citrange [35] and poplar [36]. Another category consisting in regenerating transformants from established embryogenic cultures has been described in several species including grapevine [21,37], Citrus [38], rubber tree [22], *Prunus subhirtella *[18,19] and American chestnut [23]. These procedures were shown to be efficient not only for basic research in functional genomics aimed at characterizing T-DNA insertion [21,39,40], the long-term stability of transgene expression [19] or transgene silencing [41], but also for the production of transgenic varieties. In the last ten years, embryogenic callus directly produced from leaf explants has been the most widely used tissue for *Agrobacterium*-mediated transformation in coffee [14]. Nevertheless low transformation frequencies were reported and, like for other perennial crops, the availability of embryogenic tissues suitable for genetic transformation remains one of the main bottlenecks for developing genetic transformation strategies. In this work, we demonstrate that established embryogenic cultures can be used to improve the efficiency of coffee tree transformation. Another advantage of such cultures is that, unlike other target tissues used for transformation whose availability is seasonal, embryogenic cultures are available all year round. This means transformation experiments can be planned at any time, which is a prerequisite for setting up a more efficient and faster transformation pipeline.

The reliability of GFP fluorescence to monitor transformation efficiency in coffee was confirmed by a large-scale trial aimed at regenerating fully-transformed plantlets using the hygromycin marker gene. In this trial, high frequencies of hygromycin resistant calli (82.5%) were evaluated that were similar to those of GFP expressing calli (90-93%) obtained in the same transformation conditions. In a previous work on coffee, transformation efficiency ranged from less than 1% for the recalcitrant *C. arabica *[6] to 33% for *C. canephora *[42]. The fluorescent marker GFP has a major advantage compared to other reporter genes in that it enables non-invasive detection of transformed cells without the introduction of co-factors or the destruction of the biological sample [43]. Similarly, GFP visual selection was recently used as the only marker to detect transgenic calli lines of *Hevea brasiliensis *without antibiotic pressure [44]. As previously reported [7,45], highly efficient selection of transformed coffee tissues was achieved and escapes were prevented when the antibiotic hygromycin was used in the four-month selection step.

It is noteworthy that in our study most progress in improving transformation efficiency was achieved by optimizing the production conditions of the embryogenic cultures used as target tissues for transformation rather than by optimizing the physical co-cultivation conditions. The positive effect on genetic transformation of reducing the strength of mineral salts in the proliferation medium of coffee embryogenic cultures (MS/2 or MS/4) is consistent with reports in the literature. Although it has been widely demonstrated that ions are involved in bacterial attachment to plants [46], transformation has been successfully achieved in numerous species by using half-strength or even more diluted salt solution media or solutions that specifically lack certain salts such as CaCl_2 _in the pre-culture medium [47] or co-cultivation medium [48,49]. It has been shown that during long-term culture, plant cells may lose the need for auxin and/or cytokinin to maintain active growth. This process known as 'habituation', which is common in callus cultures in some plant species such as sugarbeet [50], was described as a shift from auxo- to autotrophic state for growth regulator requirements [51]. Coffee embryogenic cultures do not require auxin or cytokinin to proliferate, although it was possible to stimulate proliferation by optimum exogenous concentrations of both growth regulators. Similarly, genetic transformation of long-term cultures was possible without auxin or cytokinin. Our results specifically revealed a very negative effect of cytokinin and a positive effect of auxin in optimizing transformation ability. Blanc et al. [22] showed that simultaneously increasing the auxin and cytokinin supply in the pre-culture medium prior to transformation of rubber tree using embryogenic cultures stimulated the development of active and fast growing cells, hence improving transformation efficiency.

Coffee embryogenic callus cultures were successfully established by sub-culturing the recently formed active embryogenic tissues every four weeks. It is widely acknowledged that a lack of regular subcultures leads embryogenic calli to lose their embryogenic potential due to cell ageing. Applying short subcultures (14-21 days) on a maintenance (i.e. proliferation) medium is an essential step in establishing long-term cultures in many perennial crops [52-56]. The process of establishing coffee embryogenic cultures leads to the development and maintenance of three morphologically different callus phenotypes with highly contrasted potential for genetic transformation. Several morphological variants with contrasted transformation potential have also been observed during the proliferation and maintenance of embryogenic calli in cotton [57]. Andrade et al. [23] highlighted great variability in the way in which embryogenic cultures proliferate and that this variability should be taken into account as it strongly affects further genetic transformation. These authors described embryogenic culture proliferation as a continuum along a developmental gradient from undifferentiated embryogenic callus through slightly more differentiated proembryogenic masses or PEMs [58] to repetitive embryogenesis at the globular embryo stage or even later [59]. Our histological studies in coffee showed that, depending the callus phenotype or the age of the embryogenic culture, either distinct cell types can co-exist, resulting in a heterogeneous proliferating callus, or only one cell type is present, forming a very homogeneous tissue.

One priority of any team involved in developing a transformation procedure is to identify the suitable target cell type. At the histological level, the yellow coffee callus with high transformation ability (transformation efficiency >90%) proved to consist of PEMs similar to those observed in the *Daucus carota *model [58,60,61] that were described as proliferating compact cell masses able to produce somatic embryos. In our study, improved transformation efficiency was also systematically associated with increased quantities of PEMs when different auxin/cytokinin balances (data not shown) and embryogenic culture ages were tested. Taken together, these results indicate that PEMs are probably the competent target tissue for coffee genetic transformation in *C. arabica*. PEMs have already been identified as suitable target cells for the transformation of several woody species for which transformation methods using embryogenic cultures have been established like *Vitis vinifera *[62], *V. rotundifolia *[37], avocado [63] and American chestnut [23]. The particular structure of PEMs could favorably influence the *Agrobacterium*-mediated transformation process. For instance in *Arabidopsis thaliana*, Sangwan et al. [64] showed that, irrespective of their origin, the competent cells were small, isodiametric with thin primary cell walls, small vacuoles, prominent nuclei and dense cytoplasm. Most of these characteristics correspond to those of coffee PEMs. The small size of PEMs associated with their looser organization increases bacterial accessibility. In addition, the high regenerative potential of this cell type is an indispensable quality for regenerating transgenic plants.

Although embryogenic callus directly regenerated on leaf explants is the most widely used tissue for coffee transformation, low transformation efficiencies (< 1%) were obtained [14]. However, the histological heterogeneity of coffee embryogenic calli has already been reported [65]. Our work confirmed the strong heterogeneity of this tissue but also revealed its low transformation-competent PEM contents. Consequently, we recommend avoiding direct use of embryogenic callus for transformation experiments and instead completing a number of preliminary proliferation cycles to increase the quantity of PEMs.

Many authors have studied the influence of ageing on transgenic lines. Indeed, as embryogenic cultures of some species can be maintained for several years without loss of embryogenic capacity, they provide a model to study the long-term expression of transgenes during *in vitro *culture and in regenerated plants [19,66]. Surprisingly, little attention has been paid to the effect of the age of embryogenic cultures used as target tissues on subsequent transformation efficiency. In our study, transformation efficiency increased markedly with the age of the coffee embryogenic culture to reach maximum at seven months of proliferation, and remained at over 90% for several months. To our knowledge, this is the first time that a strong positive effect of ageing of embryogenic cultures on their transformation competence has been demonstrated. As the regeneration capacity of coffee embryogenic cultures remained stable over two years (data not shown), this means that highly efficient and reliable regeneration of transgenic plants is possible. It has been reported that some *Vitis vinifera *genotypes produced transgenic embryo lines irrespective of 4, 8, or 12 month embryogenic culture age, whereas others produced embryo lines only from the youngest 4-month cultures [20]. However, this response was attributed to differences in genotype to maintain their regeneration potential under long-term conditions and not directly to a loss of transformation competency. The same was recently observed in *Citrus *by Dutt and Grosser [38], who compared the transformation competence of six-year-old and one-year-old embryogenic cultures. In that study, although some EGFP expression in the callus phase of the old cell line was observed, it was not possible to regenerate transgenic embryos and plants from such cells. Among the parameters we studied, the age of the culture was the most important factor in improving the efficiency of coffee transformation. The optimization of this parameter should thus be taken into account in other species for which transformation procedures using embryogenic cultures as target tissues have already been established.

*Agrobacterium*-mediated transformation often allows transgenic plants containing single copy insertions to be obtained [66]. Low transgene copy numbers have recently been reported in transformants derived from embryogenic cultures in several trees [21,23,38]. Similarly, our results in coffee showed that all the selected plants had T-DNA integrated in their genome and that almost half contained single transgene insertion sites. These results are consistent with previous observations in coffee plants transformed via *A. tumefaciens *with from one to five inserted T-DNA copies, of which 69% harbored one T-DNA copy [6]. It has been shown that transgene expression is influenced by the number of T-DNA copies and/or the integration site [67]. Even if inactivation of transgene expression can occur in plants with a single copy [68], this phenomenon is less frequent than with multiple transgene copies [69,70]. For this reason, the regeneration of a high proportion of coffee plants with a low copy number of the inserted T-DNA is important for the application of this transformation technology for both genomic purposes and breeding programs.

## Conclusions

The availability of an efficient and reliable transformation procedure in coffee tree is very important given the opportunities that will be soon available in functional genomics thanks to the genome sequencing of *C. canephora*. Moreover, in a perennial species like coffee in which selection takes a very long time, the use of genetic transformation alongside conventional breeding techniques will enable the introduction of agronomically useful traits in the best cultivated varieties in only one step. In this context, it was important to establish transformation procedures based on the use of target tissues taken from the mature tissues of elite varieties. This is the case of the process developed in the present work that will enable the routine transformation of embryogenic cultures derived from leaves selected on the mother tree and the subsequent regeneration of large quantities of transformants from numerous independent transformation events.

## Methods

### Plant material

All the studies were conducted using the genotype *C. arabica *var. Caturra. The leaf explants were collected from trees maintained in a greenhouse located at IRD (Montpellier, France). Embryogenic calli were induced as previously described [71]. Briefly, 1 cm^2 ^pieces of young leaves from the mother tree were surface sterilized and used as explants. Leaves were disinfected by immersion in 10% calcium hypochlorite solution containing 1% Tween 80 for 20 min followed by 8% calcium hypochlorite solution for 10 min and rinsed four times with sterile water. The explants were cultured for one month on MS/2 [72] 'C' callogenesis medium containing 2.26 μM 2,4-dichlorophenoxyacetic acid (2,4-D), 4.92 μM indole-3-butyric acid (IBA) and 9.84 μM isopentenyladenine (iP) allowing the production of primary callus comprising cytoplasmic meristematic cells. The leaf explants were then transferred to MS/2 'ECP' embryogenic callus production medium containing 4.52 μM 2,4-D and 17.76 μM 6-benzylaminopurine (6-BA) for 6-8 months until regeneration of embryogenic callus. Yellow embryogenic callus appeared on the necrotic primary callus. Embryogenic callus was induced in baby food jars at 27°C in the dark. All the media used in this work were supplemented with 30 g/L sucrose and their pH was adjusted to 5.7 prior to the addition of 2.8 g/L phytagel. The media were autoclaved at 120°C and 1.1 kg/cm^2 ^for 20 min, and then 25 ml of medium was added to each 100 × 15 mm Petri dish or baby food jar.

To establish the embryogenic cell suspension, embryogenic calli were transferred to 250 ml Erlenmeyer flasks at a density of 1 g/L in MS/2 'CP' liquid proliferation medium [71] with 4.52 μM 2.4-D and 4.65 μM kinetin, and shaken at 100 rpm at 27 °C in the dark. Suspension cultures of embryogenic cell aggregates were generally established after three months under such conditions.

Establishment of embryogenic callus cultures: long-term embryogenic cultures were successfully established on semi-solid medium using the embryogenic callus and by transferring the yellowish fragments collected from the upper part of embryogenic calli to fresh semi-solid 'ECP' MS/2 embryogenic callus production medium [71] with 4.5 μM 2.4D and 12 μM 6-BA solidified with 2.8 g/L phytagel once a month. The cultures were kept in baby food jars at 27°C in the dark.

### Agrobacterium strain and binary vector

The disarmed strain of *A. tumefaciens *LBA1119 carrying a binary vector (pBIN35SGFP) containing the reporter gene *GFP5 *coding for green fluorescent protein under control of the constitutive cauliflower mosaic virus (CaMV) 35S promoter was used for all experiments aimed at designing a transformation protocol. The LBA1119 strain carrying the binary vector pMDC32 [5] containing the *HPTII *gene that confers hygromycin resistance was used to regenerate transgenic plants and to validate the transformation protocol in the same culture conditions.

### Co-cultivation and decontamination

Coffee explants were maintained in their culture container, i.e. baby food jars, and immersed in 10 ml of *A. tumefaciens *suspension (OD_600 _= 0.6) for 10 min without shaking. The bacterial suspension was removed and the inoculated explants were co-cultivated at 20 °C for five days in the dark. After this period, the explants were rinsed twice with 20 ml sterile water after which 20 ml of ECP medium containing 1.2 g/L cefotaxime was added to each jar. The cultures were placed on a rotary shaker at 30 rpm for three hours. The liquid was then removed and the calli rinsed with ECP medium for 15 min. The liquid was removed and the explants were blotted on dry filter paper to remove excess bacterial solution. They were subsequently placed in Petri dishes containing ECP medium with 500 mg/L cefotaxime. From each jar, four Petri dishes were made up each containing 20 explants. The cultures were placed in the dark for four weeks at 27°C after which *GFP *reporter gene expression was assessed.

### Experiments aimed at improving the efficiency of genetic transformation

In order to improve transformation conditions, the following culture conditions were compared by assessing subsequent transformation efficiency:

1. Type of target tissue. We tested three sources of embryogenic material as target tissue. Embryogenic callus conventionally used for transformation was compared to established embryogenic cultures either in liquid medium (four-month-old embryogenic cell suspensions) or on semi-solid medium (four-month-old embryogenic callus cultures).

2. Co-cultivation conditions. Two *Agrobacterium *suspension concentrations (undiluted (OD 0.6) or diluted 1/10 by adding 'ECP' medium) and two temperatures (20°C or 27°C) were tested on four-month-old embryogenic callus cultures.

3. Proliferation conditions of the embryogenic callus cultures. The following modifications to the ECP medium were compared by assessing callus growth and transformation efficiency after three sub-cultures: auxin concentration (0, 1.8, 4.5, 9 and 18 μM 2.4D), cytokinin concentration (0, 3, 13, 23 and 32 μM 6-BA) and Macro and Microelement salt concentration (MS/4, MS/2, MS, 1.5MS).

4. Callus morphology. Different embryogenic callus phenotypes observed under the same culture conditions [yellow, whitish and gray (Figures 3A1, 3B1, 3C1)] were compared for subsequent transformation efficiency.

5. Culture ageing. Embryogenic cultures of different ages (embryogenic callus or 1, 4, 7, 9, 16 and 26-month-old embryogenic callus cultures exhibiting the same yellow phenotype) were tested for genetic transformation.

### Evaluation of transformation efficiency by GFP visualization

All the embryogenic tissues tested for genetic transformation were used to evaluate stable expression of *GFP *30 days after *Agrobacterium *inoculation. These plant tissues were screened for *GFP *expression using a Leica MZ Fluo III (optic 0.63 Zeiss) fluorescence microscope supplied with a DC 300F camera (Leica Microsystems, Welzlar, Germany) with plant GFP filter no. 3 from Leica: excitation wavelengths: 470-540 nm (BP), emission wavelengths: 525-550 nm (BP). The autofluorescence from chlorophyll was blocked using a red interference filter. Transformation efficiency was calculated as the proportion (p) of transformed calli (p = x/n), where x was the number of transformed calli exhibiting GFP and n the number of co-cultivated calli.

### Histological observations

The samples were fixed for 24 h in a solution containing 1% glutaraldehyde, 2% paraformaldehyde and 1% caffeine in a 0.2 mM phosphate buffer at pH 7.2. They were then dehydrated in a graded series of ethanol, embedded in a 7100 resin (LKB) and cut into 3 μm longitudinal sections. The sections were double-stained with PAS (periodic acid-Schiff)-NBB (Naphthol blue black). PAS specifically stains polysaccharides red (walls and starch) and NBB stains soluble and insoluble proteins blue [73,74]. Sections were observed by conventional light microscopy through a DM600 Leica microscope and photographed. Two magnification lenses were used: Lens 109 Numerical aperture = 0.30 HC PL Fluotar (Ref. Leica 11506505) and Lens 209 Numerical aperture = 0.70 HC Plan APO (Ref. Leica 1506166). Pictures were taken with a Retiga 2000R camera (G-Imaging Co.).

### Regeneration of transgenic plants

The best culture conditions were applied in a large-scale experiment to transform 560 calli from seven-month-old embryogenic cultures using the LBA1119 strain carrying the binary vector pMDC32. After decontamination, the cultures were subcultured every four weeks twice on 'R' regeneration medium [71] containing 17.76 μM 6-BA and 50 mg/L hygromycin and decreasing cefotaxime concentrations (250, 125 μg.ml^-1^) and twice on 'M' maturation medium [71] containing 1.35 μM 6-BA, 100 mg/L hygromicin and 125 mg/L cefotaxime. The other subcultures were carried out on 'M' maturation medium containing 1.35 μM 6-BA devoid of cefotaxime and hygromicin until plantlets developed. Several plants regenerated from each resistant callus. Ten months after co-cultivation, the plantlets were acclimatized in the greenhouse. During the entire regeneration process up to acclimatization, the cultures were maintained under a 14 h photoperiod (20 μmol.m^-2^s^-1 ^light intensity) at 26°C.

### PCR and Southern blot analysis of transgenic plants

Sixty putatively transformed greenhouse plants derived from independent transformation events (one plant selected at random per transformation event) and produced in the large-scale experiment were subjected to PCR analysis to detect the presence of the hygromycin resistance gene. The leaves were frozen in liquid nitrogen and DNA was extracted using a CTAB procedure [75] with modified extraction buffer (2% CTAB, 1.4 mM NaCl, 100 mM Tris HCl, 20 mM EDTA pH 0.8). The primers used for amplification of a fragment of *HPTII *gene from the pMDC32 vector were: 5'-GCCTGAACTCACCGCGACGTC-3' and 5'-GCACTGACGGTGTCGTCCAT-3' (fragment size 506 bp). PCR was carried out in a 25 μl volume containing 50 ng of leaf genomic DNA, 1 U Taq DNA polymerase, 1X buffer (Promega, Madison, USA), 0.2 mM each dNTP, 2 mM MgCl_2_, 0.2 μM each primer. Amplification was performed in a thermal cycler GeneAmp^® ^PCR system 9700, Applied Biosystem) as follows: 1 cycle of 2 min at 95°C, followed by 30 cycles of 1 min at 95°C, 1 min at 55°C, 1 min at 72°C, and a final extension of 4 min at 72°C. The reaction products were electrophoresed in 1% (w/v) agarose gels and visualized by staining with ethidium bromide.

Among the PCR-positive plants, 11 that derived from independent transformation events were chosen to perform Southern blot analysis to determine the number of transgene integration sites into the genome. Briefly, 20 μg of genomic DNA was digested with the restriction endonuclease *Eco*RI. There is no *Eco*RI restriction site on the probe sequence. DNA fragment separation by electrophoresis, transfer to nylon membrane, radioactive probe labeling and hybridization were performed as previously reported [76].

### Data processing

Transformation efficiency was calculated as the proportion (p) of transformed calli (p = x/n), where x was the number of transformed calli exhibiting GFP and n the number of co-cultivated calli. A 3 δ confidence limit for binomial distribution was calculated using the formula  with a level of confidence of 99%. The transformation experiments with different types of target tissues were conducted independently in three replicates each containing 50 to 80 calli (150 to 240 tissues/type of target tissue) and experiments with different co-cultivation parameters in 3 or 4 replicates each comprising 26 to 85 calli (80 to 350 calli/co-cultivation parameter). The experiments with different mineral concentrations were conducted independently in four replicates each comprising 22 to 40 calli (87 to 160 calli/mineral concentration). The experiments with different hormone combinations were conducted independently in four replicates each comprising 60 calli (240 calli/ hormonal combination). For each callus phenotype, all the transformation experiments were conducted independently in three replicates each comprising 40 calli (120 calli/phenotype). For each culture age, all the transformation experiments were conducted in five to six replicates each comprising 40 calli (200 to 240 calli/culture age). Callus growth (mg/month) was measured by the difference between the final and initial weight of the embryogenic cultures after a 1 month proliferation cycle. The initial weight was calibrated at 120 ± 10 mg. Each datum corresponds to the mean ± SD of 4 measurements. We performed an ANOVA followed by the Tukey HSD test to identify significant differences between the means of all treatments.

## List of abbreviations

6-BA: 6-benzylaminopurine; CaMV: cauliflower mosaic virus; CP medium: callus proliferation medium; 2,4-D: 2,4-dichlorophenoxyacetic acid; ECP medium: embryogenic callus production medium; GFP: green fluorescence protein; HPTII: hygromycin phosphotransferase; M medium: maturation medium: OD: optical density; PEMs: proembryogenic masses; R medium: regeneration medium; SD: standard deviation; TAIR: the Arabidopsis information resources.

## Authors' contributions

AFR conducted most of the transformation experiments, performed transgenic plant regeneration and the PCR and Southern analyses, and took part in data analysis, and was centrally involved in writing the manuscript. ED carried out most of the transformation experiments. AC took part in writing the manuscript. BB performed the statistical analyses and was involved in writing the manuscript. MCC participated in the Southern blot experiments. JLV took part in analyzing the histological data and FL carried out the histological studies. PL took part in drafting the manuscript. HE designed the overall project, analyzed the results, planned and took part in the experiments, and was a primary author of the manuscript. All authors read and approved the final manuscript.
